# Mendelian Randomization Analysis of Human Blood Metabolites and Sensorineural Hearing Loss

**DOI:** 10.1002/brb3.71577

**Published:** 2026-07-15

**Authors:** Xingxing Ye, Wenbao Wu, Jiaqian Dai, Yurong Ye, Yinjuan Chen, Yangui Chen, Daofeng Fan

**Affiliations:** ^1^ Department of Otorhinolaryngology Longyan Second Hospital Longyan Fujian China; ^2^ Department of Acupuncture and Moxibustion Longyan First Hospital Affiliated to Fujian Medical University Longyan Fujian China; ^3^ Department of Neurology Longyan First Hospital Affiliated to Fujian Medical University Longyan Fujian China

**Keywords:** blood metabolites, lipid metabolism, Mendelian randomization, sensorineural hearing loss

## Abstract

**Objectives:**

The role of the human blood metabolites and sensorineural hearing loss (SNHL) has been extensively studied, but the exact causal relationship remains uncertain. To address this knowledge gap, two‐sample Mendelian randomization (MR) analysis was employed to investigate the potential causal effects of blood metabolites on the risk of developing SNHL.

**Methods:**

Genetic associations with SNHL were mainly derived from FinnGen (cases: 44,745; controls: 437,331), while human blood metabolites were extracted from met‐a, met‐d, and 1400 blood metabolites. Inverse variance weighted (IVW) and other MR methods, such as MR‐Egger, weighted median, simple mode, MR‐PRESSO and Steiger Test were used to explore the causal relationship between blood.

**Results:**

In the met‐d cohort, a causal association was identified between four lipid metabolites and SNHL. Specifically, free cholesterol‐to‐total lipids ratio in small very‐low‐density lipoprotein (VLDL) (IVW OR = 0.95, 95% CI = 0.92–0.99, FDR *p* = 0.01), free cholesterol‐to‐total lipids ratio in medium VLDL (IVW OR = 0.93, 95% CI = 0.90–0.97, FDR *p = *0.00), and cholesterol‐to‐total lipids ratio in large high‐density lipoprotein (HDL) (IVW OR = 0.94, 95% CI = 0.91–0.98, FDR *p* = 0.00) were significantly associated with a reduced risk of SNHL. Furthermore, the average diameter of VLDL particles (IVW OR = 1.06, 95% CI = 1.01–1.11, FDR *p* = 0.01) was significantly associated with an increased risk of SNHL. In contrast, within the met‐a cohort and across the 1400 blood metabolites analyzed, no blood metabolites were identified to be associated with SNHL. After conducting the Steiger Test, we did not find any reverse causality.

**Conclusions:**

Our MR Study provides support for the genetic evidence of a causal connection between blood metabolites and SNHL. Additionally, it offers new insights into the exploration of strategies for the prevention and management of SNHL.

## 1 Introduction

Sensorineural hearing loss (SNHL) has emerged as a significant societal health concern within contemporary society. The global burden of disease study reveals that hearing loss ranks as the fourth leading cause of disability on a global scale (GBD 2015 Disease and Injury Incidence and Prevalence Collaborators 2016). SNHL can engender challenges in communication and impede interpersonal relationships, thereby precipitating various forms of depression and anxiety. Although the mortality rate directly attributable to this affliction remains low, its profound influence on overall quality of life is undeniable. Consequently, the prevention and treatment of SNHL have assumed paramount importance within current research endeavors (Z.‐Q. Zhang et al. 2023). Hearing loss is primarily ascribed to the impairment of endocochlear lesions. Exposure to noise levels surpassing a certain threshold can result in various damages to cochlear structures, encompassing the stellaria, hair cells, supporting cells, and even the mantle membrane. These damages may potentially be influenced by metabolite processes (Wan et al. 2014). The field of otolaryngology is currently witnessing substantial advancements through the utilization of a cutting‐edge technique called metabolites, particularly in the context of SNHL (Noto et al. 2022). The adoption of this methodology will lead to modifications in the diagnosis and treatment of specific otolaryngological diseases. Metabolites, a systems biology approach, seeks to explore the etiology and diagnostic indicators of clinical ailments (Guo et al. 2022). Previous research has demonstrated that the heritability of hearing loss varies between 25% and 55%, with over 100 genes identified as harboring mutations responsible for hearing impairment, in addition to approximately 30 mutations linked to progressive hearing loss (Lin et al. 2023; Christensen et al. 2001). Hence, the utilization of metabolite analysis is imperative in investigating the pathological mechanisms underlying SNHL, thereby enhancing the efficacy of preventive and therapeutic interventions.

The validation of the association between blood metabolites and SNHL represents a pressing concern. Randomized controlled trials (RCTs) are regarded as the benchmark for establishing causality in epidemiological research. Nevertheless, the execution of such trials is hindered by ethical limitations and substantial financial burdens. Conversely, observational studies are frequently employed for initial causal exploration owing to their less complex design and ease of implementation. However, it is important to acknowledge that these studies frequently encounter limitations, including the presence of confounding factors and the potential for reverse causality. In order to address these challenges, the implementation of Mendelian randomization (MR) offers a valuable methodology that employs genetic variation as an instrumental variable (IV) to investigate the causal association between exposure and outcome. By doing so, MR effectively mitigates the impact of confounding factors and the influence of reverse causality (Davies et al. 2018; Burgess et al. 2015). Consequently, genotypes can be utilized as IV to thoroughly examine intermediate phenotypes, thereby facilitating the inference of causality in relation to disease conditions (Bowden and Holmes 2019). This methodology successfully mitigates the influence of confounding variables and reverse causation.

However, the precise role of blood metabolites in mediating the mechanism of SNHL remains uncertain, underscoring the significance of investigating the causal relationship between blood metabolites and SNHL. We put forward the following hypothesis: blood metabolites—such as lipid metabolism, amino acids, and purine‐related compounds—play a causally influential role in the onset of SNHL. Employing MR as an analytical framework, we hypothesize that these blood metabolites are not merely associated with SNHL, but actively contribute to its pathogenesis. Validating this causal link may uncover new molecular targets for intervention and guide the development of more precise, mechanism‐driven therapeutic approaches for affected individuals.

## 2 Materials and Methods

### 2.1 Ethics Statement

This investigation was conducted using publicly available Genome‐Wide Association Study (GWAS) summary statistics. All original studies contributing data had received ethical clearance from their respective institutional review boards, and written informed consent was obtained from all participants prior to enrollment. As our analysis is based solely on de‐identified, aggregate‐level genetic data, no further ethics review or participant consent was required.

### 2.2 MR Assumptions

**FIGURE 1:**
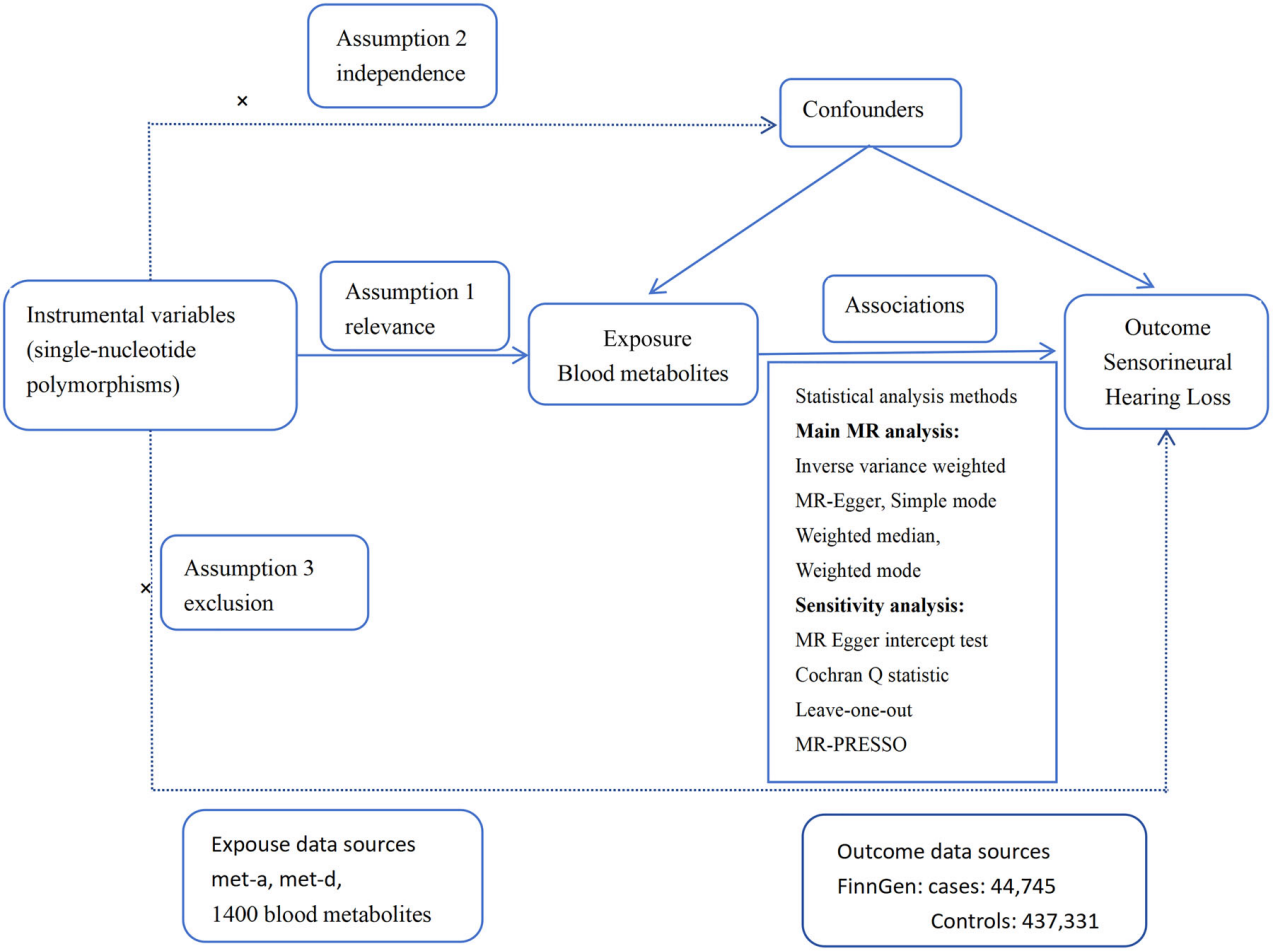
Dashed lines indicate potential pleiotropic or direct causal effects between variables that may violate MR assumptions. MR‐PRESSO, Mendelian randomization pleiotropy residual sum and outlier.

The MR analysis is based on three fundamental assumptions: relevance, independence, and exclusion (Emdin et al. 2017). The selected genetic variations are hypothesized to have a substantial impact on the risk factor, but they do not demonstrate any association with other influencing factors in the relationship between the risk factor and outcome. Additionally, these genetic variations are not connected to the outcome through any alternative pathway apart from the risk factor of interest. Figure 1 depicts the general layout of the research.

### 2.3 Instrumental Variable Selection

In the study, a rigorous threshold of *p* < 5 × 10^−8^/5 × 10^−6^ was initially established to meticulously identify single‐nucleotide polymorphisms (SNPs) exhibiting significant associations with SNHL and the blood metabolites. Additionally, to address potential linkage disequilibrium (LD), these SNPs were effectively grouped with a 10,000 kb limit and an *r* ^2^ = 0.001 threshold. When certain SNPs surpassed the LD threshold of 0.1, the blood metabolites associated SNPs with the smallest *p* value were selected for subsequent analysis. If there were no blood metabolite–related SNPs available in the resulting dataset, proxy SNPs (with an *r* ^2^ value greater than 0.8) were selected for MR analysis using the 1000 Genome European Reference Panel. Subsequently, the phenotypic variance of each blood metabolite, which can be attributed to the corresponding genetic variation, was calculated using the two‐sample MR software. To ensure reliable causal inference with sufficient statistical power, metabolite variations explained by genetic factors less than 0.5% were excluded from the analysis (Chong et al. 2019). Furthermore, in order to ensure robustness, specific MR correlation analyses were conducted, excluding metabolite traits that had fewer than three associated SNPs across the entire genome (Cho et al. 2020).

### 2.4 Data Source for Blood Metabolites and SNHL

Aggregated data pertaining to genetic variation linked to the human blood metabolites were acquired from three extensive GWAS, encompassing a collective sample size of 122,902 individuals of European ancestry. As per the investigation conducted by Shin et al. (2014), a comprehensive analysis of metabolites was carried out on 7824 participants, scrutinizing 453 metabolite traits (met‐a) and approximately 3 million SNPs within this study. Julkunen H. et al. (UK Biobank 2023) conducted a comprehensive analysis of metabolic signatures (met‐d: 249 blood metabolites) in a large cohort of 115,078 participants from the UK Biobank. This analysis involved the examination of approximately 12 million SNPs using the Nightingale health analysis method. Furthermore, we incorporated plasma metabolome data derived from four distinct population cohorts, with a total of 1400 metabolites identified. The European cohort comprised 8299 participants, while the South Asian, East Asian, and African cohorts included 108, 104, and 60 individuals, respectively. GWAS were performed on all samples, encompassing approximately 15.4 million SNPs loci. The associated data were retrieved from the GWAS database of the European Bioinformatics Institute (https://www.ebi.ac.uk/gwas/). Specifically, the GWAS summary statistics for the European population are accessible via accession numbers GCST90199621–GCST90201020, whereas the data for the South Asian, East Asian, and African populations are collectively archived under the series identifiers GCST90201021–GCST90204063 (Chen et al. 2023). Furthermore, the R12 release of the Finngen database (https://finngen.gitbook.io/documentation/) provides data on 44,745 patients with SNHL and 437,331 control subjects.

### 2.5 Statistical Analysis

The Inverse variance weighting (IVW) method is widely acknowledged as the most statistically robust approach in MR analysis, as it provides consistent estimates of causal effects when all genetic variants are valid instrumental variables. Nevertheless, the IVW method relies on the assumption that all genetic variants satisfy the exclusion restriction criterion—that is, they influence the outcome solely through the exposure of interest. This assumption may not always hold in practical applications due to the potential presence of horizontal pleiotropy (Burgess et al. 2013). To address this concern and enhance the reliability of causal inference, we incorporated supplementary instrumental variables to systematically examine the association between the protected exposure and the outcome. Specifically, in addition to the IVW method, we employed the weighted median (WM) method (Bowden et al. 2016) and the MR‐Egger method (Bowden et al. 2015) for sensitivity analysis. The WM method offers a dependable evaluation of the causal impact between exposure and outcome, provided that valid instrumental variables contribute a minimum of 50% of the weight in the analysis (Bowden et al. 2016). This approach remains robust even when up to half of the genetic variants are invalid due to pleiotropic effects (Bowden et al. 2016). On the other hand, the MR‐Egger method is capable of identifying and correcting for horizontal pleiotropy by estimating an intercept term that quantifies the average pleiotropic effect across all genetic variants. However, it is important to note that MR‐Egger tends to have lower precision and wider confidence intervals compared to IVW, and its estimates may be less accurate, particularly when there is substantial heterogeneity or when the InSIDE (instrument strength independent of direct effect) assumption is violated. Heterogeneity among the individual variant‐specific causal estimates was evaluated using Cochran's Q statistic derived from the IVW analysis, where a significant Q statistic would indicate the presence of heterogeneity possibly due to pleiotropy or other model misspecifications. To formally address horizontal pleiotropy at the directional level, we calculated the *p* value of the MR‐Egger model intercept; a statistically significant intercept (*p* < 0.05) would suggest the presence of directional horizontal pleiotropy, thereby supporting the use of MR‐Egger over IVW. The estimation of the F‐statistic is conducted using aggregated data. If the *F*‐value surpasses 10, it is presumed that the correlation is sufficiently robust to counteract any potential bias arising from a less influential independent variable. To ascertain the overall causal effects, we implemented a leave‐one‐out sensitivity analysis technique, systematically removing one SNP at each interaction. In the event that MR‐Pleiotropy Residual Sum and Outlier (MR‐PRESSO) identifies any outlier SNPs, these data points were initially excluded from the analysis. Afterwards, the remaining instrumental variables were subjected to additional evaluation in order to ascertain the most suitable statistical approach (Verbanck et al. 2018). Once the recommended methodology was identified, we proceeded to perform sensitivity analyses concurrently employing alternative analytical techniques. Moreover, we utilized the MR Steiger approach to ascertain the direction of causation (Hemani et al. 2017). In order to ensure the reliability of our findings, we implemented Bonferroni's adjustment to address multiple testing, setting a threshold at < 0.000024 (0.05/2101 = 0.000024) (Georgakis et al. 2019). In this research, a *p* value below 0.000024 (FDR‐*p* < 0.05) was deemed as significant evidence indicating a causal relationship between exposure and the outcomes. The statistical analysis was conducted utilizing the two‐Sample MR package (Cho et al. 2020), MR, and MR‐PRESSO in R (version 4.3.1).

## 3 Results

### 3.1 Genetically Determined Met‐d and SNHL

**FIGURE 2:**
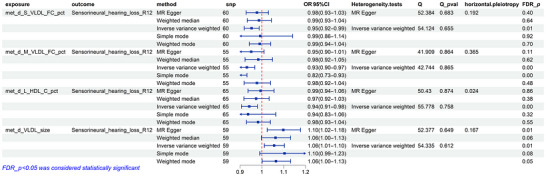
Genetically determined met‐d and SNHL. CI = confidence interval; met_d_S_VLDL_FC_pct = free cholesterol‐to‐total lipids ratio in small VLDL; met_d_M_VLDL_FC_pct = free cholesterol‐to‐total lipids ratio in medium VLDL; met_d_L_HDL_C_pct = cholesterol‐to‐total lipids ratio in HDL; met_d_VLDL_size = average diameter of VLDL particles; OR = odds ratio; Q = Cochran's Q test; snp = single nucleotide polymorphisms.

IVW‐MR analysis demonstrated a significant negative association between the genetically predicted levels of three blood metabolites in met‐d and SNHL (all correlation *p* values were < 0.05 following Bonferroni correction). Free cholesterol‐to‐total lipids ratio in small very‐low‐density lipoprotein (VLDL) (IVW OR = 0.95, 95% CI = 0.92–0.99, FDR *p* = 0.01), free cholesterol‐to‐total lipids ratio in medium VLDL (IVW OR = 0.93, 95% CI = 0.90–0.97, FDR *p* = 0.00), and cholesterol‐to‐total lipids ratio in large high‐density lipoprotein (HDL) (IVW OR = 0.94, 95% CI = 0.91–0.98, FDR *p* = 0.00) were significantly associated with a reduced risk of SNHL, where an OR < 1 indicates that each one‐unit reduction in the genetically predicted metabolites level ratio is associated with a corresponding decrease in the risk of SNHL. Our study further revealed a statistically significant association between the average diameter for VLDL particles (IVW OR = 1.06, 95% CI = 1.01–1.11, FDR *p* = 0.01) and susceptibility to SNHL. This suggests that each unit increase in the genetically predicted average diameter for VLDL particles corresponds to a 6% elevation in the likelihood of developing SNHL. An FDR *p* value < 0.05 denotes statistical significance (Figure 2).

**FIGURE 3:**
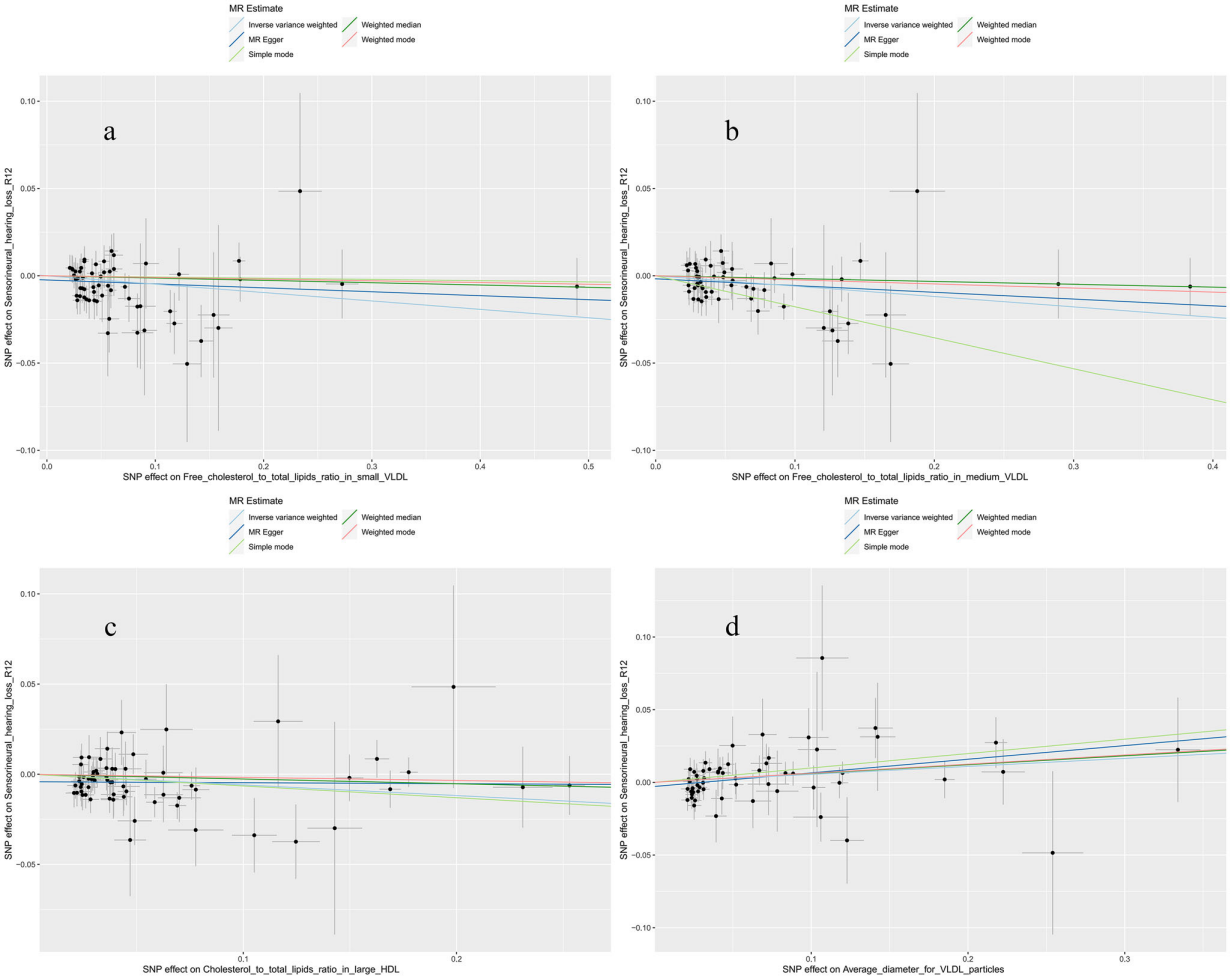
Scatter plot of met‐d and SNHL. (a) Free cholesterol‐to‐total lipids ratio in small VLDL, (b) free cholesterol‐to‐total lipids ratio in medium VLDL, (c) cholesterol‐to‐total lipids ratio in HDL, and (d) average diameter of VLDL particles.

**FIGURE 4:**
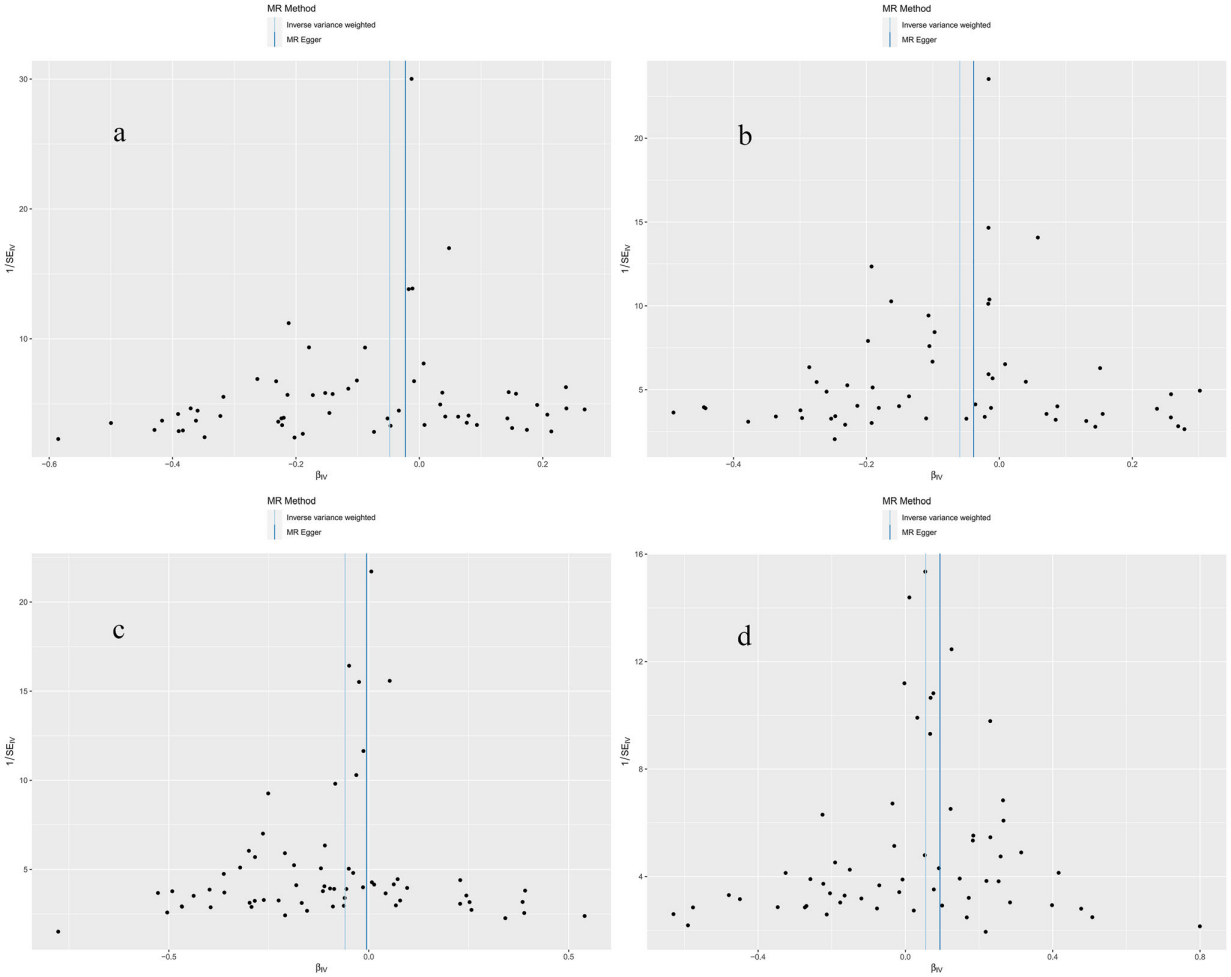
Funnel of met‐d and SNHL. (a) Free cholesterol‐to‐total lipids ratio in small VLDL, (b) free cholesterol‐to‐total lipids ratio in medium VLDL, (c) cholesterol‐to‐total lipids ratio in HDL, and (d) average diameter of VLDL particles.

**FIGURE 5:**
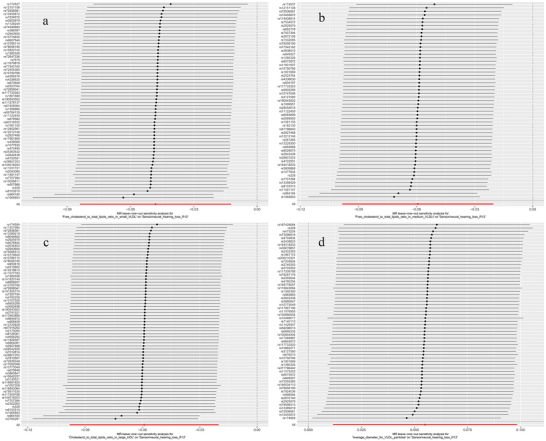
Leave one out of met‐d and SNHL. (a) Free cholesterol‐to‐total lipids ratio in small VLDL, (b) free cholesterol‐to‐total lipids ratio in medium VLDL, (c) cholesterol‐to‐total lipids ratio in HDL, and (d) average diameter of VLDL particles.

**FIGURE 6:**
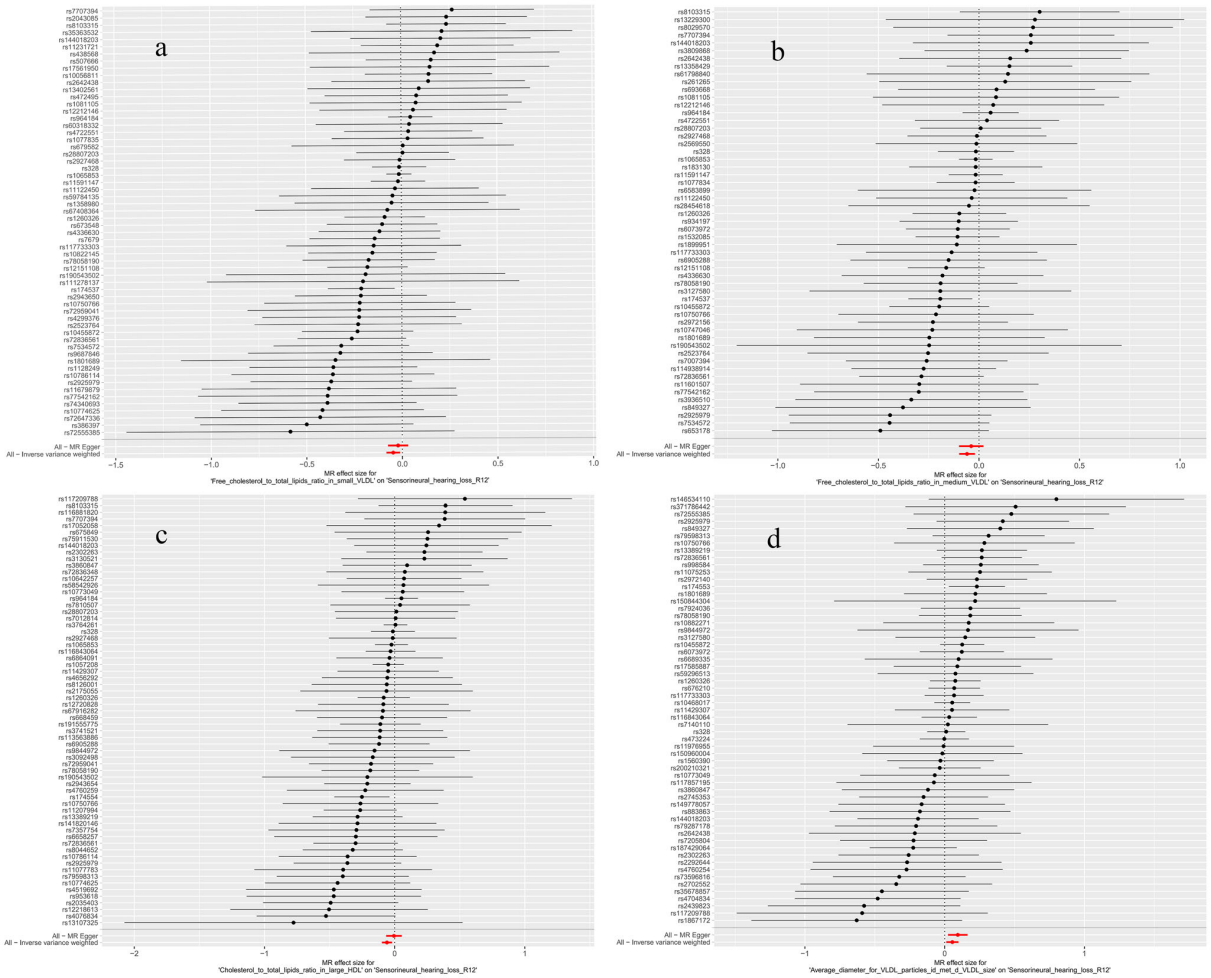
Forest of met‐d and SNHL. (a) Free cholesterol‐to‐total lipids ratio in small VLDL, (b) free cholesterol‐to‐total lipids ratio in medium VLDL, (c) cholesterol‐to‐total lipids ratio in HDL, and (d) average diameter of VLDL particles.

The scatter plots, funnel plots, leave‐one‐out analysis and Forest plots sensitivity analyses of MR investigations for blood metabolism on SNHL are presented in Figures 3, 4, 5, 6.

All SNPS used in the analysis were included in Supporting Information S1.

### 3.2 Effects of Met‐a and 1400 Metabolites on SNHL

This study employed the IVW method for MR analysis. We found no evidence of causal effects of Met‐a or any of the 1400 blood metabolites on SNHL, as all corresponding IVW *p* values exceeded the conventional significance threshold of 0.05.

### 3.3 Reliability Assessment of Results

#### 3.3.1 Heterogeneity Testing

Cochran's Q test, performed separately for MR‐Egger and IVW estimates, indicated no statistically significant heterogeneity (all *p* > 0.05) in the causal associations between the four met‐d‐derived blood metabolites within the met‐d subgroup and SNHL: free cholesterol‐to‐total lipids ratio in small VLDL (MR‐Egger = 52.384; IVW ≤ 54.124), free cholesterol‐to‐total lipids ratio in medium VLDL (MR‐Egger = 41.909; IVW ≤ 42.744), cholesterol‐to‐total lipids ratio in large HDL (MR‐Egger = 50.43; IVW ≤ 55.778), and average diameter for VLDL particles (MR‐Egger = 52.377; IVW ≤ 54.335) (Figure 2).

#### 3.3.2 Sensitivity Testing

To evaluate the robustness of our causal estimates, we performed multiple sensitivity analyses—specifically, the WM method and leave‐one‐out tests—following IV construction based on genome‐wide significant SNPs. Concordant results across these complementary methods lend strong support to the validity and stability of the MR findings (Figures 2 and 5).

#### 3.3.3 Horizontal Pleiotropy Testing

MR‐Egger regression was employed to perform an intercept test aimed at evaluating potential horizontal pleiotropy. In the MR analyses, the intercept *p* values for free cholesterol‐to‐total lipids ratio in small VLDL (0.19), free cholesterol‐to‐total lipids ratio in medium VLDL (0.36), and average diameter for VLDL particles (0.17) all exceeded 0.05, implying negligible bias from horizontal pleiotropy attributable to genetic variants. However, the intercept *p* value for the cholesterol‐to‐total lipids ratio in large HDL was below 0.05, suggesting that the horizontal pleiotropy bias attributable to genetic variants warrants attention.

### 3.4 Steiger Test

Steiger testing indicated no evidence of reverse causation: all *p* values for the associations between the following lipoprotein traits—namely, the free cholesterol‐to‐total lipids ratio in small and medium VLDL, the cholesterol‐to‐total lipids ratio in large HDL, and the mean VLDL particle diameter and SNHL—were reported as zero, supporting the hypothesized direction of effect.

## 4 Discussion

With the continuous growth of the elderly population, SNHL will pose a serious threat to human health and has become one of the major public diseases in the world, and it is becoming increasingly important to identify and develop potential protective or therapeutic approaches. In this study, MR method was used to analyze the role of blood metabolites (met‐a, met‐d, and 1400 blood metabolites) on SNHL, providing theoretical basis for further investigation of the pathogenesis of SNHL. Assuming adherence to the fundamental IV conditions (sufficient strength, unconfoundedness, and no horizontal pleiotropy), causal estimates were derived primarily via the IVW method. Our results identified three lipid metabolism (met‐d) significantly linked to lower SNHL risk: the free cholesterol‐to‐total lipids ratio in small VLDL (IVW OR = 0.95, 95% CI = 0.92–0.99, FDR *p* = 0.01), free cholesterol‐to‐total lipids ratio in medium VLDL (IVW OR = 0.93, 95% CI = 0.90–0.97, FDR *p* = 0.001), and cholesterol‐to‐total lipids ratio in large high‐density lipoprotein (HDL) (IVW OR = 0.94, 95% CI = 0.91–0.98, FDR *p* = 0.001). Conversely, the larger average VLDL particle diameter was associated with higher SNHL susceptibility (OR = 1.06; 95% CI: 1.01–1.11; FDR *p* = 0.01). Notably, no statistically significant associations were observed between any of the met‐a, 1,400 blood metabolites and SNHL—after multiple testing correction. Additionally, Steiger filtering provided no evidence of reverse causation.

It is important to note that lipid metabolism plays a vital role in SNHL. A comprehensive meta‐analysis conducted by Rodrigo et al. (2021) encompassing 22 studies demonstrated that diets high in cholesterol and unsaturated fatty acids had adverse effects on hearing. Conversely, diets rich in polyunsaturated fatty acids were found to have a protective effect against hearing loss. Deng Bangyu et al. (Deng et al. 2025) confirmed through a two‐sample MR study that triglycerides (TG) have a significant causal effect on SNHL, significantly increasing the risk of SNHL. However, this study only analyzed the levels of three lipid indicators in routine biochemical tests and has not yet explored the association between lipid metabolites and SNHL. Luo et al. (2026) found that an elevated ratio of ApoB/ApoA is associated with poor prognosis of SNHL, while HDL‐C is an independent influencing factor for a good prognosis of SNHL. Meanwhile, Sharma et al. (2023) demonstrated that serum total cholesterol and TG levels are significantly positively correlated with the degree of hearing loss; HDL levels show a negative correlation trend with the severity of hearing loss. Lipid profiles can serve as important biomarkers for assessing the severity of hearing loss—subjects with abnormal lipid parameters have more severe hearing impairment. Yin Ji Yuan et al.’s study based on the 1999–2016 National Health and Nutrition Examination Survey (NHANES) data in the United States indicated that residual cholesterol is associated with adverse hearing outcomes (including hearing loss and tinnitus), with a clear dose‐response relationship pattern that remains stable after adjusting for lipid levels on both sides (Yin et al. 2026). While their findings further elucidate the association between conventional lipid levels and SNHL, discrepancies remain between their results and those of the present study. The current study focused on the analysis of lipid metabolites, identifying four lipid metabolites associated with SNHL: three were found to reduce the risk of SNHL onset, whereas one was associated with an increased risk.

Considering the impact of lipid metabolites on SNHL is reasonable from multiple perspectives. Firstly, the inner ear receives its blood supply from a singular labyrinthine artery, and disturbances in lipid metabolism can contribute to the development of sclerosis in this artery. Consequently, inner ear hypoperfusion and cochlear microvascular ischemia may ensue, culminating in sensory hair cell injury and, ultimately, SNHL (Papadopoulou et al. 2024; Kaneva et al. 2019). However, impairment to the blood supply of the cochlea can impede the cellular metabolism of waste removal, hinder the maintenance of cochlear potential, disrupt ion transport, and disturb the balance of lymphatic fluid in the blood vessels that maintain the integrity of the blood labyrinth barrier (Shi 2009). Additionally, lipids play a crucial role in the peripheral nerve sheath, and any abnormalities in lipid metabolism can have a significant impact on sudden hearing loss (Eckhardt 2023; Yamamoto et al. 2017). The relationship between lipid metabolism and hearing levels appears to be more intricate than previously hypothesized, and may be contingent upon the quantity of fat subclasses ingested. Thus, a comprehensive investigation into how lipid metabolism contributes to the pathogenesis of SNHL is critically warranted. Furthermore, it is crucial to acknowledge the contribution of other metabolites in SNHL to effectively mitigate and minimize the incidence of sensory deafness.

Metabolomics has emerged as a robust tool for examining disease phenotypes, providing a wealth of data for the detection of biomarkers, comprehension of pathogenesis, and customization of therapeutic approaches (Johnson et al. 2016). Numerous investigators have employed metabolomics to investigate individuals affected by hearing loss with the aim of elucidating the association between these variables (Huang et al. 2018; X. Zhang et al. 2023). Fujita et al. conducted a study that revealed that guinea pigs exposed to high levels of noise experienced notable modifications in the composition of the inner ear fluid. Specifically, a total of 10 metabolites demonstrated significant changes, encompassing amino acid decomposition metabolites and lipid compounds (Fujita et al. 2015). Meanwhile, Miao et al. (Wang et al. 2021) conducted a lipid metabolomics investigation involving 60 patients with HL and 60 individuals with normal hearing. Their findings indicated a significant correlation between HL and metabolic pathways such as glycerophospholipid metabolism, choline metabolism, linolenic acid metabolism, and linoleic acid metabolism. This study utilized a two‐sample MR approach to evaluate the causal relationship between met‐a and 1400 metabolites and SNHL. However, our results demonstrated no significant association between either met‐a or the 1400 metabolites and SNHL. While prior studies have explored the association between human blood metabolites and SNHL, a more comprehensive and systematic investigation is still required to fully elucidate the causal relationship between the two.

In contrast to traditional observational studies, our specialized approach (MR Study) eliminates the influence of confounding factors, and we employed the MR‐Steiger technique to establish causality. Secondly, we performed multiple sensitivity analyses to confirm the robustness of the main findings. This magnetic resonance study represents a pioneering attempt to assess the causal relationship between blood metabolites and SNHL. However, it is crucial to acknowledge certain limitations. Specifically, due to data limitations, our investigation solely focused on the correlation between blood metabolites and SNHL, disregarding the potential association with other types of hearing impairments. Moreover, it is imperative to recognize the potential presence of racial bias given that the study participants were exclusively of European ancestry. As a result, a prudent approach is warranted when generalizing these findings to other racial groups. Additional research is indispensable to validate our findings and explore their potential implications in clinical interventions.

## 5 Conclusion

In conclusion, our MR Study provides empirical support for the genetic evidence establishing a causal relationship between blood metabolites and SNHL. These findings present new avenues for investigating strategies aimed at preventing and managing SNHL.

## Author Contributions

**Wenbao Wu**: writing – original draft, funding acquisition, methodology, software. **Jiaqian Dai**: writing – review and editing, validation, visualization, software. **Yurong Ye**: validation, visualization, software. **Yinjuan Chen**: visualization, supervision. **Xingxing Ye**: methodology, software, supervision, conceptualization, visualization, funding acquisition, writing – original draft. **Yangui Chen**: writing – review and editing, data curation, visualization, supervision. **Daofeng Fan**: writing – original draft, conceptualization, funding acquisition, project administration, methodology.

## Funding

This research was financially supported by Longyan City Science and Technology Plan Project (Grants 2024LYF17096, 2023LYF17043, and 2023LYF17044), Fujian Provincial Natural Science Foundation (Grant No. 2023J011879) and The Longyan Neurorehabilitation Medical Science and Technology Team.

## Ethics Statement

This meta‐regression analysis employed previously published summary statistics from GWAS. The respective institutional review boards' ethics committees granted written informed consent to all participants in the original studies. Consequently, no further ethical approval or informed consent is necessary for conducting this analysis.

## Consent

The authors have nothing to report

## Conflicts of Interest

The authors declare no conflicts of interest.

## Supporting information




**Supplementary Materials**: brb371577‐sup‐0001‐SuppMat.xls

## Data Availability

The present study relied on publicly available summary statistics from genetic consortia's genome‐wide association studies as its primary data source. Specifically, the data utilized in this study were obtained from Open GWAS, accessible at (https://gwas.mrcieu.ac.uk/).
